# Role of Vitamin C in Osteoporosis Development and Treatment—A Literature Review

**DOI:** 10.3390/nu12082394

**Published:** 2020-08-10

**Authors:** Olga Brzezińska, Zuzanna Łukasik, Joanna Makowska, Konrad Walczak

**Affiliations:** 1 Department of Rheumatology, Medical University of Lodz, 92-115 Lodz, Poland; zuzanna.lukasik@stud.umed.lodz.pl (Z.Ł.); joanna.makowska@umed.lodz.pl (J.M.); 2 Department of Internal Medicine and Nephrodiabetology, Medical University of Lodz, 90-050 Lodz, Poland; konrad.walczak@skwam.lodz.pl

**Keywords:** vitamin C, ascorbic acid, nutrition, osteoporosis, bone loss, diet

## Abstract

Osteoporosis and associated low energy fractures are a significant clinical problem, especially in the elderly population. The occurrence of a hip fracture is associated with significant mortality and a high risk of disability. For this, apart from the treatment of osteoporosis, effective prevention of both the development of the disease and related fractures is extremely important. One aspect of osteoporosis prevention is proper dietary calcium intake and normal vitamin D3 levels. However, there is some evidence for a potential role of vitamin C in osteoporosis and fracture prevention, too. This review aims to summarize the current knowledge about the role of vitamin C in osteoporosis development, prevention and treatment. The PubMed/Medline search on the role of vitamin C in bone metabolism database was performed for articles between 2000 and May 2020. Reports from in vitro and animal studies seem promising. Epidemiological studies also indicate the positive effect of high vitamin C content in the daily diet on bone mineral density. Despite promising observations, there are still few observational and intervention studies and their results do not allow for unequivocal determination of the benefits of high daily intake of vitamin C or its long-term supplementation.

## 1. Introduction

The term “osteoporosis” in its current meaning was most probably used for the first time by a French pathologist Jean Frederic Lobstein the Younger in 1830. The word derived from the Greek *osteon* (bone) and *poros* (little hole). At the beginning it was used to describe cavities observed in human bone during autopsy. In the 20th and 21st century new definitions incorporating disease pathogenesis, diagnostic measures and complications were created [1]. Despite constantly growing clinical experience, osteoporosis remains a major cause of morbidity and mortality in the elderly population. According to a World Health Organization scientific group report, osteoporotic fractures account for 2.8 million disabilities in the Americas and Europe [2]. The lifetime risk of a wrist, hip or vertebral fracture has been estimated between 30% and 40% in developed countries, what makes it close to the risk of coronary heart disease. In the European Union, the number of deaths related to osteoporotic fractures was estimated to be 43,000 in 2010 [3].

Diet and lifestyle-associated variables constitute the most significant risk factors for osteoporosis development. Among these, inadequate nutritional absorption, dietary deficiencies, obesity, lack of physical activity and smoking are listed [4]. These factors are not only potentially modifiable, but also strongly interdependent. Multiple studies have proven the crucial role of calcium and vitamin D on bone homeostasis. In the guidelines on osteoporosis management issued by the European Society for Clinical and Economic Aspects of Osteoporosis and the International Osteoporosis Foundation, precise recommendations on daily calcium and vitamin D intake have been defined [5]. The importance of other nutrients on preserving high bone mineral density has been suggested and pathways in which these compounds affect bone metabolism are being investigated [6]. Furthermore, dietary choices influence the composition of gut microbiota, a complex network of microorganisms capable of metabolizing foodstuffs into metabolically active compounds influencing the host homeostasis. The gut microbial composition of primary osteoporosis patients has recently been characterized and was found to differ significantly from healthy age- and sex-matching controls [7].

Although the symptoms of osteoporosis can already be seen in the skeletons of ancient human beings [8] and the first clinical descriptions appeared in the XIX century, we are still enriching our knowledge about the aetiology of the disease, the cellular mechanisms that govern it, and effective methods of treatment and prevention. Currently available therapeutic interventions in osteoporosis include calcium and vitamin D supplementation, as well as novel antiresorptive drugs such as ibandronate and denosumab. Despite their clinically proven efficacy, primary prevention is the mainstay of osteoporosis management. Several risk factors for the development of osteoporosis have been identified, pointing towards the role of genetics and biological factors, as well as environmental factors, including diet. Dietary composition may favour the disease or protect against it. The mechanisms linking the diet and bone metabolism have not been fully elucidated. One plausible explanation is the putative buffering effect of acids derived from vegetables and fruits [8,9]. Moreover, numerous epidemiological studies suggested the importance of micronutrients such as ascorbic acid (vitamin C, VC) in protection against bone loss, not only due to their buffering effects [10,11,12]. Vitamin C is an organic chemical compound from the group of unsaturated polyhydroxy alcohols. It is a water-soluble nutrient synthesized by all plants and most animals, except humans [13,14]. Overdosing of VC can lead to mild symptoms such as osmotic diarrhoea and nausea, since the excess is excreted with urine [13]. Ascorbic acid deficiency leads to a well described set of symptoms known as scurvy. The syndrome includes several bone symptoms: osteolysis, osteonecrosis, bone loss and pathological fractures [8,15,16]. Nowadays not only is antioxidant function of VC suggested, but it has a role as an important mediator in bone metabolism, regulation of gene expression or activation of immune system; enzyme co-factors are known, too [14,17].

This review aims to summarize the current knowledge on the role of VC in osteoporosis development, prevention and treatment. The recent understanding of its molecular role in disease aetiology as well as potential clinical benefits of ascorbic acid supplementation are presented.

## 2. Materials and Methods

**Figure 1:**
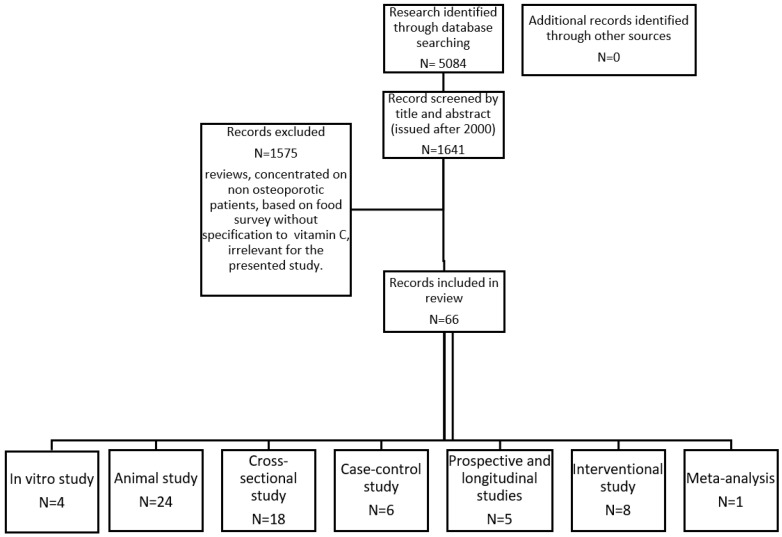
PRISMA (Preferred Reporting Items for Systematic Reviews and Meta-Analyses) flowchart showing the selection criteria used to identify studies with the search strategy. N—Number.

To provide comprehensive information about the role of vitamin C in the pathogenesis, prophylaxis and the therapy of osteoporosis, the results of both in vitro and animal model results as well as observational and intervention studies in humans have been included in this review. The review includes scientific publications published between January 2000 and May 2020 and accessible via the PubMed/Medline database. The following keywords were used as search criteria: “vitamin C,” “ascorbic acid,” “dietary,” “antioxidant,” “nutrient,” “bone metabolism,” “osteoblast,” “osteoclast,” “osteoporosis” and “bone loss.” There was no restriction on language or research group characteristics. The search results were screened for relevance. Finally, 66 articles were included in the review; 29 of them were observational studies on humans and the next 8 were interventional ones. In addition, 28 descriptions of animal and in vitro tests are presented (Figure 1).

This review presents the results of animal and cell line studies followed by cross-sectional, case-control, long-term observational and intervention studies discussed with growing clinical credibility.

## 3. Results

### 3.1. Molecular Function of Vitamin C in Bone Tissue

Vitamin C has the chemical formula C_6_H_8_O_6_ and it is purely the L-enantiomer of ascorbate. As previously mentioned, most animals are capable of de novo synthesis of VC from uridine diphosphate (UDP) glucose, but several species including the guinea pig, capybara and humans are lacking the terminal enzyme in ascorbate biosynthetic pathway [18]. Early cell-free, in vitro experiments have demonstrated the most prominent role of VC as an antioxidant [19]. Intracellularly, VC acts as a reducing agent, free radical scavenger, crucial enzyme cofactor [20] and co-substrate. It maintains the activity of ubiquitously expressed enzymes involved in collagen hydroxylation, amidation of peptide hormones, regulation of hypoxia-inducible factor and histone demethylation [14]. All known physiological effects of VC are assigned to its chemical property of a hydrogen ion and electrons donor.

#### 3.1.1. Studies on Animal Models

Several animal models have been used to elucidate the role of vitamin C in bone metabolism. The observation of guinea pigs has appointed the bone tissue as the second most vulnerable to VC deficiency organ [21], which was associated with improper collagen synthesis. Severe vitamin C deficiency inhibited animal growth [22] and was associated with a significant change in collagen composition, namely deoxypyridinoline and the pyridinoline: eoxypyridinoline ratio in the femur shaft [23]. Bone mineral content and density has also been shown to decrease in scorbutic guinea pigs, paired by a thinner growth plate and larger osteoclast surface and impaired collagen synthesis at articular cartilage and tendons [24]. Since rats and mice are capable of synthesising ascorbate, transgenic animals fed a VC-depleted diet have been designed to study skeletal effects of vitamin C deficiency. The skeletal effects of genetic transformation can be countered by oral vitamin C supplementation, underpinning the actual significance of ascorbic acid deficiency in these models. One such approach is to deplete rodents of the final enzyme in the vitamin C biosynthesis pathway, namely l-gulono-γ-lactoneoxidase, which is non-functional in humans. The characteristic skeletal features of these animals include spontaneous fractures, reduction of the cortical zone and trabecular bone volume. The trabecular bone reduction is most prominent in the close proximity of the growth plate, pointing towards the impairment [25] of new bone production in the vitamin C deficiency state [26]. This morphological observation is matched by biochemical indices of deficient osteogenesis, namely reduced levels of osteocalcin [26]. Furthermore, bone mineral density in animals with deleted genes for l-gulono-γ-lactoneoxidase is reduced by even 70% in comparison to wild-type animals [27]. Other mouse models are based on the depletion of different enzymes involved in the vitamin C biosynthesis pathway, i.e., aldolase and aldehyde reductase and gulonolactonase [25]. This results in varying degrees of VC deficiency, with only mildly reduced production in the case of aldolase reductase knockout (KO) animals and abrogated synthesis in double KO mice. Importantly, these mice not only exhibit the abovementioned skeletal features, but also decreased numbers of osteoblasts [28]. This effect is particularly prominent in the senescence marker protein (SMP) 30 KO mice, characterized by an upregulation of osteoclast differentiation markers [29,30].

Because osteoporosis commonly affects women in the post-menopausal age, ovariectomized rodents are operative models for studying its pathogenesis and therapeutic interventions [31]. Interestingly, oral vitamin C supplementation following ovariectomy in mice was shown to be protective against bone loss and osteoblast depletion [32,33]. Vitamin C intake resulted in an increased expression of osteoblast differentiation genes in time [32], suggesting that vitamin C could stimulate bone formation by inducing maturity and mineralizing osteoblast phenotype. The same group has shown that VC prevented bone resorption by osteoclasts in inflammatory milieu, pointing towards the plausible efficiency of vitamin C supplementation as a therapeutic intervention in both postmenopausal osteoporosis and bone loss associated with chronic inflammatory conditions [34]. VC deficiency was also linked to increased expression of tumour necrosis factor ligand superfamily member 11 (RANKL), which acts as a critical osteoclast and bone resorption inducer, but also an inflammatory mediator [29]. A recent study has demonstrated that ostoblastogenesis and osteoclastogenesis in osteoporosis rat models are in fact regulated by a shared serine/threonine kinase and mitogen-activated protein kinase (MAPK) signalling pathway, which is fine-tuned by vitamin C [35].

#### 3.1.2. In Vitro Models

These findings are supported by in vitro studies. Vitamin C supplementation induced the differentiation of primary bovine osteoblasts and resulted in an increased synthesis of extracellular matrix collagen, osteonectin and osteocalcin [36]. A similar effect, dependent on VC-enhanced collagen synthesis, was observed for human osteoblast-like cells [37]. Ascorbic acid alone was sufficient to induce osteoblast differentiation from human mononuclear cells isolated from peripheral blood [38] and bone mouse marrow-derived stromal cells [39]. VC supplementation in a spontaneous fracture mouse model was recently shown to induce promoter regions of osteoblast differentiation genes [40]. This finding stands in line with another proven mechanism of VC induction of osteoblastogenesis, where osteoblast differentiation regulator osterix (Osx) is upregulated in bone marrow stromal cells upon vitamin C intake and causes the nuclear factor-E2-related factor-1 (Nrf1) to bind an antioxidant-responsive element (ARE) in the Osx promoter [41].

Studies on the influence of vitamin C on chondrocyte populations brought ambiguous effects. Early in vitro experiments have shown an induction of chondrocyte differentiation and increase in extracellular matrix production [42]. Later cell culture-based studies demonstrated a dose-dependent proapoptotic effect of VC on human articular chondrocytes [42]. In oxidative stress conditions, VC protected the articular chondrocyte from damage and stimulated extracellular matrix production, but at the same time inhibited the differentiation of chondrocytes [43]. The VC-enhanced matrix formation was found to further mediate the differentiation of the chondrogenic cell line [44]. Prolyl Hydroxylase Domain-Containing Protein 2 (Phd2) has been identified as a mediator of both cartilage and bone effects of vitamin C via regulating osterix expression of osteoblasts and the hypoxia-inducible factor 1α (HIF1α) pathway [45,46]. Pgd2 is highly expressed in both chondrocytes and osteoblasts and was discovered to hydroxylate HIF1α, which enables its degradation. This promotes osteoblast differentiation [46] but suppresses chondrocyte differentiation, which is dependent on HIF1α downstream target genes [40,41,47].

Surprisingly, in vitro studies reported both suppressive and enhancing effects [25,48] of vitamin C supplementation on osteoclastogenesis. An elegant study of mouse bone marrow cultures explained this discrepancy, suggesting that ascorbic acid might play a dual role in the differentiation and survival of osteoclasts. VC was suggested to first act as an oxidant, and higher oxidative stress levels were shown to be associated with osteoclast differentiation, but at a later stage it induced osteoclast death [49].

### 3.2. Cross-Sectional Studies

Seventeen cross-sectional studies published since 2000 have been identified (Table 1). Most data compared bone mineral density (BMD) measured at different locations, mainly lumbar spine and femoral neck. Two publications assessed the risk of osteoporotic fractures, and a few research groups included bone turnover markers in their analysis. The results are extremely difficult to compare due to a large variety of markers chosen by the authors. The total number of patients included in all the studies presented reaches almost 43,000 from ten different countries, however no African, Eastern and Western European, Central, Eastern and Southeastern Asian and Central American countries are represented. The vast majority of researchers focused their attention on women in the postmenopausal period [50,51,52,53,54,55,56,57,58,59,60,61,62,63,64,65,66,67,68] and only six studies [50,52,61,64,65,67] included male participants. Three studies evaluated younger participants, too [59,65,67].

The supplementation of VC was discussed only in two of the identified studies [56,62]. Long term (2–10 years) regular supplementation of ascorbic acid was correlated with a lower concentration of C-telopeptide which is the serum bone resorption marker, while the relation with alkaline phosphatase (AP) concentration and BMD results was not significant [56]; the data about supplementation dosage was not shown. In high dosage of ascorbic acid (≥1000 mg/d), the higher BMD of ultra-distal and midshaft radii, hip and lumbar spine were observed, and only the last one was not statistically significant [62]. A positive association indicates that higher dietary VC intake was related to higher BMD in post-menopausal women [50,51,53,54,55,58,59,61,63] but several other groups reported no significant relationship between the two parameters [56,57,60,64,65,66,67,68]. This result was again confirmed in a multifactorial analysis of VC status and bone mass density in pre- and postmenopausal women [65]. Higher VC intake, understood as ascorbic acid supplementation, was associated with higher BMD in premenopausal women. No correlation was found between the serum level of VC and BMD or prevalence of self-reported fractures in this group. In the group of postmenopausal women, factors strongly influencing bone and VC metabolism—smoking and hormonal replacement therapy with the use of estrogens—were included in the model. After stratification for these factors, in the group of non-smokers and non-users of estrogens, serum VC level showed an inverse association with BMD. In the group of smoking estrogen users, increasing ascorbic acid levels were associated with a lower fracture risk [65].

Usually, positive observations concern femoral neck and lumbar spine BMD. A few studies also included men in whom daily VC intake in the diet was reported as slightly lower than in women [52,64,67] or higher [61,65] depending on the studied population. Serum VC concentration has been shown to correlate with low energy fracture risk [52,65] and BMD level [61,65,67], but lack of correlation was reported, too [64,67]. A positive impact of dietary VC intake on BMD may be confirmed by the cumulative result obtained from the meta-analysis in which BMD at the lumbar spine (pooled r = 0.15; 95% CI, 0.09–0.23) and BMD at the femoral neck (pooled r = 0.20; 95% CI, 0.11–0.34; *p* < 0.05) showed beneficial effects of dietary intake of vitamin C oriented food [12]. Discrepancies between the results obtained may result, among others, from the differences in protocols of the conducted tests and the exclusion criteria used.

Most of the presented research was based on the assessment of the average daily intake of vitamin C from data obtained via different types of Food Frequency Questionnaires. These are standardized, semiquantitative surveys created as a tool for assessing habitual intake through a detailed description of foods and drinks typically consumed. There are currently over 200 different forms available in the literature, the size of which ranges from 5–350 items [69]. The researchers of the presented study used forms containing 79–220 items [12,53,54,56,57,58,61,63,64,66,68,69,70,71,72,73,74,75,76,77] as well as 24-h dietary recall [50,51,55,59,65,76], 3–7 day diet diary [52,58,60,67,77] and other surveys dedicated to their study [63]. The diversity of the methodology used to assess the average daily dose of vitamin C may cause difficulties in comparing the results obtained. Various exclusion criteria were used; some studies took into account chronic diseases, while others did not.

Smoking has been suggested as an important factor influencing bone metabolism and VC adsorption from the gastrointestinal tract. The differences reported between the cohorts could result from different smoking status of study participants, which was not always considered in the analysis, and in some studies was an exclusion factor. It has been shown that vitamin C levels in the smoker population are significantly lower than in non-smokers, regardless of the type of diet followed [78]. Hormone replacement therapy probably does not change the serum vitamin C level [79], however, it significantly affects the change in bone metabolism. Ascorbic acid alone cannot replace the documented benefits of oestrogen replacement therapy and calcium supplements, but it appears to have an additive effect [62]. Similarly, it seems that for a more reliable assessment of vitamin C metabolic activity it is necessary to correct the results obtained to the results of calcium, vitamin D and serum parathyroid hormone levels. Failure to consider the above data may significantly interfere with the results obtained. It is worth noting that most of the cross-sectional studies did not include an element of laboratory tests, which limits the inference out of the data obtained from the survey on nutrition, habits, chronic diseases, etc. The dependence on vitamin D3 levels on the geographical location of the inhabited country can significantly affect the differences between the observed results.

Only a few of the presented studies assessed the relationship between vitamin C intake and the risk of low-energy fractures. Their results indicate a beneficial effect of higher VC intake on the reduction of fracture risk, however the relationship was observed only in men [52,65]. In a meta-analysis prepared by Zeng et al. [12] it was concluded that patients with a higher frequency of vitamin C intake had a 34% (95% CI, 6–53%) lower prevalence of hip fracture.

Few observational studies focused on a younger population. The beneficial effect of VC on BMD was observed in a group of boys aged between 16 and 18 years old in the spine size-adjusted BMD measurements [67]. In premenopausal women, a higher BMD was measured in calcaneus and total femoral region measurements [59,65] but not in the total body BMD [66].

A considerable variety in the research methodology used both in terms of recruitment of the test group, selected endpoints and the methods of their assessment may be the reason for the discrepancies observed between the studies. Most of them, however, indicate a potentially positive impact. Probably more studies with a wider laboratory stratification would be necessary to obtain more clinically reliable observations. In addition, cross-sectional studies should always be treated as an epidemiological study revealing potential relationships without being able to prove them during such a designed study. Long-term observations in well-characterized cohorts and intervention studies are needed. The data from them are presented below.

### 3.3. Case-Control Study

The case-control study type seems to have an advantage over the similar cross-sectional design, involving a retrospective search for disease development risk factors starting from the selected endpoint, in this case osteoporosis. However, it does not make the analysis of the sequence of events possible, which significantly reduces their role in providing the evidence of causality. In the available literature from the last 20 years, only five case-control studies were identified (summarized in Table 2). The purpose of these studies was to determine the relationship between dietary vitamin C intake [71,75,76,80] or its serum concentration [71,76,81] and the risk of osteoporosis [75,81] or low-energy fractures [71,76,80].

Of the six studies discussed, three evaluated the relationship between vitamin C and osteoporotic fracture. In two of them the protective effect of ascorbic acid was demonstrated. In the study of Martínez-Ramírez et al. [71] there was no difference in daily intake of vitamin C in the diet between groups, but a lower serum concentration was observed in fracture patients. In addition, for both dietary intake and serum levels, the lowest quartile (<203 mg/d and ≤1.48 mg/L, respectively) of vitamin C was associated with the highest risk of fracture. A similar result was obtained in the study of Sun et al. [79], but lower daily VC intake was demonstrated in the examined group and no serum VC concentration was determined. In both of the above studies, vitamin C intake was assessed on the basis of FFQ, however, with a different number of items.

In contrast to the above, Lumbers et al. [77] evaluated both serum level and daily vitamin C intake (24-h dietary recall) in patients with hip fracture and non-fracture age- and sex-matching control groups. In the fracture group the level of ascorbic acid was higher (serum 7.52 ± 3.77 mg/d, dietary 60.7 ± 33.2 mg/d) than in the control group (serum 3.66 ± 2.50, *p* < 0.001; dietary 55.2 ± 38.8 mg/d non-significant). A possible cause of discordant observation in comparison with two previous studies can be the fact that different schemes of food habit data collection were used, and the control group was recruited from day centers. Moreover, in the whole research group the daily intake was extremely low and, as compared to other studies, it was contained in the first and second quartile. However, the authors associate a high level of vitamin C in the serum of patients with hip fracture with the presence of orange juice in the hospital menu. This makes it difficult to assess the credibility of the obtained result. In addition, the time which has elapsed since the fracture to the recruitment was different (two weeks vs. more than six months). Matínez-Ramírez’s team postulates the necessity of a minimum six months of a grace period from the time of the fracture before qualifying patients for the study due to potential changes in vitamin C plasma levels associated with ongoing inflammation and healing at the site of the injury [71].

The other two authors qualified postmenopausal women who were diagnosed with osteoporosis—the Maggio team [81] considered the cut-off point T-score ≤−3.5, while Park et al. used a standard T-score ≤−2.5 [75]. Both studies showed a positive correlation between femoral neck BMD and daily vitamin C intake. There was also a significantly lower risk of developing osteoporosis in patients with high vitamin C intake (136.9–176.3 mg/d) compared to the lowest intake (≤91.5 mg/d) [75] and a significantly lower serum vitamin C concentration (5.28 ± 0.65 vs. 9.77 ± 2.31 mg/L; *p* <0.001) [81].

### 3.4. Longitudinal and Prospective Studies

The third type of research presented is the long-term prospective observation. Thanks to several time points at which unified data are obtained from the study group, they provide a more reliable source of information on the relationship between the exposure to environmental factors and the disease development. Among the available literature of the last 20 years, we were able to identify only five long-term studies (Table 3). The follow-up period ranged from 2 to 17 years and included a total of over three thousand patients, mainly postmenopausal women. In all but one publication [77] the evaluation of ingredients provided in the daily diet was made based on the standard semi-quantitative FFQ questionnaire.

Only Sahni et al. chose low-energy hip or other non-vertebral fractures as the endpoint of the study. They presented the results of a 15–17-year observation of 918 older women and men group. In the observed group, 100 hip and 180 other fractures located outside the spine were reported. In the analysis, the protective trend in the correlation was observed between dietary VC and risk of hip as well as non-vertebral fracture. A significantly lower risk of both types of osteoporotic fracture was observed in the highest tertile of summary VC intake (median > 300 mg/d) as well as in the group with high dosage supplementation (median 260 mg/d). The BMD at femoral neck was measured in a baseline and used for the correction in the final statistical analysis. In the rest of the presented studies, the BMD changes were the equivalent of a hard endpoint and only two of them included men in the research group [72,77]. The first of the presented studies [77] included 470 men in the seventh and eighth decade of their life (average age 72). The nutritional habits were estimated using 7-day food diaries and the lifestyle and anthropometric variables such as smoking, physical activity and BMI were taken into consideration in data analysis as well. For the male study group no relationship between VC daily intake and BMD loss in the total hip was observed.

Sahni et al. included a similar age group of men (average age 75) in their observations [72]. This study, with a similar average follow-up of four years, showed significantly lower bone loss in the lumbar spine and distal femur in the group with the highest vitamin C intake. However, it was pointed out that this effect is only visible in the population of male smokers with a low intake of calcium and vitamin E. The results of both studies can be regarded as complementary, although it is difficult to compare them even though they relate to a similar age group, observation time and the same sex. The other elements of the study design differ significantly—in the study by Kaptoge et al., 7-day food diaries were used to assess the composition of the diet and total hip BMD was used as the endpoint, whereas in Sahni’s observation, a standard semi-quantitative FFQ was used to assess nutrient intake, the first BMD measurement was performed using a dual-photon absorptiometer at femoral neck and lumbar spine locations, and X-ray absorptiometry was used in the follow-up. The results obtained in the baseline were calculated to enable their comparison between the methods. Although the authors indicate that the X-ray absorptiometer measures were lower than the dual-photon absorptiometer measures [72,83], this does not allow for a simple comparison of the results and may affect their quality and size of the observed effect.

Both of the abovementioned cohorts also included a female population. The results obtained again seem to be in contrast because in Sahni’s [72] study there was no correlation between daily VC intake and change in BMD. In the population described by Kaptoge et al. [84], in turn, a significantly greater bone loss was found in the group with the lowest daily intake of vitamin C in the diet compared to medium and high intake, respectively −0.65%, −0.31% and −0.30% per annum. The other two observations not discussed above include only women. The first small group of 187 post-menopausal women was based on a nutritional survey (no more detailed information about its structure), serum concentration of carotenoids and densitometry of non-dominant forearm performed at baseline and after four years of follow-up [70]. As a result of this study, a statistically significant reduction of osteoporosis development risk (OR = 0.16 95% Cl 0.03–0.95) was shown in a group with high VC intake and high serum levels of β-cryptoxanthin. No correlation was observed in other tested groups with high VC and low β-cryptoxanthin as well as low CV and low or high β-cryptoxanthin. Unfortunately, the baseline study group was limited, so only 15 cases of newly developed osteoporosis were observed after four years, whereas in the group with both high intake of VC and serum β-cryptoxanthin there were only two cases. Moreover, the statistical importance of lower odds ratio disappeared after adjustments for calcium, magnesium, potassium and vitamin D. It is difficult to assess whether the obtained outcome resulted from the lack of relationship between the studied nutrients and the risk of developing osteoporosis or was associated with observed groups that were too small.

The last study we identified was also the only one whose participants were middle-aged (45–55 years old) at the start of the study [74]. This Scottish cohort gathered 891 women who underwent lumbar spine densitometry and femoral neck and assessed nutritious habits through FFQ. In the course of the data analysis, numerous associations of nutrients such as fatty acids, retinol and VE with a change in bone density were found. In premenopausal women, a positive correlation was demonstrated between bone density within the femoral neck and the observed change in this BMD, in both cases food energy-adjusted.

Although the number of patients under observation in most of the presented studies is close to a thousand, there are still too small groups and too short observations to gain a fully reliable picture of the connection between vitamin C level taken with a diet and, additionally, supplementation with the risk of developing osteoporosis and low-energy fractures. The information on young people is particularly limited, because only one of the presented works included middle-aged women, while the role of VC was included only as the secondary information. In addition, the availability of information on men is limited and there is no observational study identified for middle-aged or younger men.

### 3.5. Interventional Studies

Interventional studies are the only ones that allow the determination of the clinical benefits of using a drug or a dietary supplement in the treatment or prevention of disease development; in this case, the determination of the usefulness of ascorbic acid supplementation in the prevention and treatment of osteoporosis. However, few such studies have been carried out in the last twenty years (Table 4). Randomized, double-blind, placebo-controlled studies are considered to be the most reliable. To our knowledge, there are only two studies with randomization and double-blind design and two with randomization only.

The first randomized double-blind study conducted in Mexico by the team of Ruiz-Ramos et al. gathered 90 elderly subjects (both men and women), who were then divided into three groups: one group receiving placebo and two supplementing vitamin E (400 IU) and 500 or 1000 mg of VC, respectively, for 12 months [82]. Chronic diseases and smoking status were not used as excluding factors; everyday nutritional habits and serum VC and VE concentration were not controlled. The statistically significant smaller hip bone loss was observed in the group with highest VC intake versus placebo control and there was no similar relation in lumbar spine density.

The second double-blind trial contained 33 elderly male participants randomly divided into two groups: strength training program (three sessions/week) connected with placebo or VC 1000 mg and VE 235 mg supplementation [85]. The participants were healthy, without significant disability, not using steroids or antioxidant supplementation, and not training systematically before the inclusion. The dietary habits were evaluated by four-day diary registration. Significant BMD proliferation of the lumbar spine was reported in the placebo group compared to those who additionally were supplemented with antioxidants. The increase of total hip BMD was observed only in the placebo group, no changes in the femoral neck and total body density were noted in both groups. It appears that vitamin C and E supplementation limits the positive effect of resistance training on bone remodelling. However, the study was carried out for a short time (12 weeks) and in a small group of volunteers, and neither smoking status nor primary VC serum levels were considered. In a similarly projected study [86] with a longer, six-month follow-up, no similar restrictive effect of antioxidant supplementation to benefit from systematic resistance training was seen. No difference was observed between the groups with antioxidant supplementation, systematic physical activity and the connection of both interventions. In all of the above cases the stabilization of the lumbar spine BMD was observed while in the placebo group a significant decrease in BMD was noted. Similar results of ascorbic acid connecting with vitamin E, selenium and alpha-lipoic acid in Mainini et al.’s [87] study or vitamin B6 and proline in Masse et al. [73] were shown. In both studies the placebo group experienced bone loss while the intervention was connected with mixed results—a discreet increase in bone mass [88] or deterred bone loss [87].

In the other presented clinical trials only laboratory markers of bone metabolism were taken into consideration. The first of them was the study with a design similar to Stunes [86] and Chuin [88] in which the strength training program was one of the tested interventions. The Maïmoun team [68] pilot study recruited nine elderly women and four men to an 8-week experiment with VC (500 mg) and VE (100 mg) supplementation connected with supervised, one-hour training three times a week. The serum concentration of vitamin D, calcium, parathormone and bone metabolism factors were measured. After two months, a significant increase in vitamin D serum level and bone alkaline phosphatase was observed, which suggests the improvement of the calciotropic hormone profile. Unfortunately, in this study the BMD was not evaluated, so the clinical effect is difficult to assess.

In the case of two intervention studies [83,89] conducted so far, the effect of VC supplementation as monotherapy was assessed. In both cases, 500 mg ascorbic acid was supplemented for 12 and 13 weeks, however, it was not placebo-controlled. In the study of Bjarnson et al. [83], both VC supplementation and its combination with fluvastatin did not cause significant changes in the studied bone turnover markers (including alkaline phosphatase). A different result was obtained in the observation of Chavan’s team [90], in which a significant decrease in the concentration of alkaline phosphatase as well as tartrate resistant acid phosphatase, malondialdehyde, calcium ions, glutathione reductase and superoxide dismutase were noted. The effect was observed both in the group supplemented with vitamin E alone (except for the decrease in erythrocyte which reduced glutathione and alkaline phosphatase levels) and vitamin C alone and both antioxidants at the same time. The observed results may indicate both a decrease in osteoclast activity and the regulation of mechanisms preventing oxidative stress, which is difficult to refer directly to bone turnover mechanisms.

Due to the wide variety of protocols for intervention studies, it is difficult to compare the results obtained in them. Out of the currently available studies, in three the intervention time was one year, the others were significantly shorter; moreover, only small groups were observed (the largest trial included 90 participants [82]). None of the studies compared VC to placebo; only in two [83,89] was it used in monotherapy. The effect of supplementation is also difficult to determine in the group of younger people who were the subject of observation only in Masses et al.’s study. So far there has been no clear evidence for the benefits from systematic VC supplementation, however, there are premises suggesting its beneficial effect. However, it is necessary to conduct a long-term study on a large group of both young and elderly people and assess the individual effect of ascorbic acid supplementation.

## 4. Conclusions

The impact of nutrition on health is an extremely appealing topic. Dietary compounds delivered to the body every day have a significant impact on the body functions by regulating its metabism and providing the necessary nutrients for the proper development and functioning of the organism.

There are many in vitro and animal model studies that confirm the significant influence of vitamin C on the skeletal system. Its postulated multi-directional effects range from the inhibition of osteoclast activity and stimulation of osteoblast maturation by increasing collagen type I synthesis to the regulation of gene transcription, DNA and histone methylation. The clinical picture of severe vitamin C deficiency in the form of scurvy is also beyond doubt and has already been described in ancient times. However, the evidence for a beneficial effect of vitamin C supplementation in people without a significant deficiency is not so clear. A large variety of conducted clinical trials, small groups of patients, variable follow-up time and supplementation schedules make it difficult to assess the effectiveness unambiguously. In addition, some of the intervention studies did not bring the expected results in the form of bone mass increase. The data available so far do not allow an unequivocal assessment of the usefulness of vitamin C supplementation in the prevention and treatment of osteoporosis. However, it seems that a proper content of VC in the diet has a beneficial effect on bone metabolism, so it is worth paying attention to the advantageous impact of a proper diet rich in products containing vitamin C. This is part of the current recommendation for consuming five servings of vegetables and fruit a day. At the moment, the clinical efficiency of ascorbic acid supplementation remains controversial. Recommending the patients to follow a proper, well-balanced diet seems to be the most appropriate approach. A long-term observation on a wide population and prolonged interventional studies following a unified study design in different patient subgroups are required to draw unequivocal conclusions.

## Figures and Tables

**Table 1:** Cross-sectional studies assessing the association between dietary vitamin C intake or vitamin C serum concentration and BMD or low energy fracture.

Study	Study Population	Dietary Assessment	Outcome Measure(s) and Lab Analyses	Results
New SA et al., 2000 [78] UK	62 healthy women pre- and postmenopausal (range 45–54 years old)	FFQ	LS and FN BMD Quantitative CT at the ultradistal radial total, trabecular, and cortical sites Urine concentration of pyridinoline and deoxypyridinoline Serum level of osteocalcin	Daily VC intake: 103.4 ± 65.6 mg/d (23.8–453.2). Mean deoxypyridinoline excretion was significantly lower with higher intakes of VC (*p* < 0.02). No correlation with BMD parameters in any localisation.
Simon JA et al., 2001 [65] USA	3778 premenopausal women (34 ± 8 years old) 3165 postmenopausal women (62 ± 13 years old) 6137 men (44 ± 16 years old) 20–90 years old	24-h dietary recall	FN BMD Serum level of VC and VE Self-reported fractures	In a premenopausal women group, increasing levels of dietary VC were independently associated with BMD and did not correlate with prevalence of self-reported fractures. In a postmenopausal women group, neither VC intake nor VC serum concentration was associated with BMD and or prevalence of self-reported fractures. Among postmenopausal women who had no history of smoking or oestrogen use, serum ascorbic acid levels were inversely associated with BMD. In smoking postmenopausal women with history of oestrogen use, higher VC supplementation was associated with a lower prevalence of fractures.
Ilich JZ et al., 2003 [58] USA	136 postmenopausal (at least 5 years) women, generally health, 68.7 ± 7.1 years old	3-day dietary record (2 weeks and 1 weekend day); Ca-FFQ	TB, LS, FN, forearm and hand BMD Serum levels of PTH and vitamin D	A weak but statistically significant correlation between VC dietary intake and bone mass was observed. VC intake was a significant element of stepwise regression models explaining loss of Ward’s triangle, trochanter, shaft and total femur BMD.
Wolf RL et al., 2005 [63] USA	11,068 postmenopausal women aged 50–79 years old	FFQ 122 food items supplements were estimated with an interviewer-administered questionnaire	TB, LS, TH BMD Serum concentrations of retinol, carotenoids, and tocopherols measured (N = 379)	The beneficial effect of current hormone therapy use on FN TB, LS and TH BMD was greater in women with higher VC level. An independent positive influence of VC intake on BMD was not observed.
Prynne CJ et al., 2006 [67] UK	Three groups: 101 girls and 111 boys 16–18 years old 90 women 23–37 years old and 67 women and 67 men 60–83 years old	7-day food diaries	TB, LS, TH, FN and greater trochanter BMD; bone area and bone mineral content	In the group of boys, significant positive associations were found between dietary VC and BMD. A negative association between hip BMD and VC intake was found in the group of older women.
Pasco JA et al., 2006 [56] Australia	533 postmenopausal, non-smoking women 48–89 year old (mean 68.3) 26 supplemented vitamin C and 22 vitamin C and/or E	Ca-FFQ Self-reported supplement and medication use, diet, and lifestyle questionnaire	TB BMD Serum bone resorption markers: C-telopeptide and bone-specific AP	Lower concentration of C-telopeptide was observed in group with increased exposure to VC and/or VE.
Sugiura M et al., 2011 [54] Japan	293 postmenopausal women	FFQ 97 food items	BMD of nondominant forearm	High intake of VC (170–625 mg/day) with β-cryptoxanthin was inversely associated with low radial BMD and may be beneficial to bone health.
Rivas A et al., 2012 [59] Spain	280 women divided into 3 groups: ≤35, 35–45 and ≥45 years old	24-h diet recall	BMD of calcaneus	BMD was higher in the participants defined as high antioxidant consumers in all age groups.
De França NA et al., 2013 [60] Brazil	150 postmenopausal women, 68.7 ± 9.1 year old (range 48–87 year old)	3-day food diary, non-consecutive days	TB, LS, FN and TF BMD	No relationship between the BMD and daily antioxidant intake was found.
Karamati M et al., 2014 [57] Iran	151 postmenopausal women aged 60.3 years old	FFQ 168 food item	LS and FN BMD	Mean BMD of the lumbar spine of women in the highest tertile of the first pattern contain folate, total fiber, potassium, vitamin A, C, K, B6, b-carotene, magnesium, copper, and manganese scores was significantly higher than those in the lowest tertile (mean difference 0.08; 95% confidence interval 0.02–0.15; *p* = 0.01). No correlation between separate nutrients or nutrient patterns and BMD was found.
Kim YA et al., 2015 [51] South Korea	1196 postmenopausal female	24-h dietary recall	LS, FN and TH BMD	Dietary vitamin C intake tertile was significantly positively associated with BMD at all sites (R = 0.513 for LS and R = 0.657 for FN; *p* < 0.05 for each). The multiple-adjusted odds ratio for osteoporosis for dietary VC <100 mg/day was 1.790 (95% CI 1.333–2.405; *p* < 0.001). However, a significant association between VC intake and BMD was only observed in subjects with vitamin D deficiency and aged 50–59 years or >70 years.
Finck H et al., 2015 [52] UK	4510 participants (women: 2616, men 1898), the mean age 60 ± 10 years old	7-day diet diary	History of bone fractures VC plasma concentration	A linear inverse relationship between quintiles of plasma VC and the prevalence of hip fractures (HR: 0.82; P-trend = 0.016) in men.
Liu ZM et al., 2015 [61] China	2000 men and 2000 women aged 65 years and older 72.5 ± 5.2 years old	FFQ 266 food items	BMD, bone mineral content, and bone area at TB, TH, LS and FN	TB and FN BMD were significantly positively associated with fruit intake in both men and women. Adjustment for VC intake, but not dietary acid load, attenuated the association between fruit intake and bone mass.
Kim MH et al., 2015 [55] South Korea	1467 postmenopausal women Age 65.2 ± 0.3 years old	24-h recall	TF, FN and LS BMD	Participants consuming less VC than the estimated average requirement showed higher odds (OR = 1.49; 95% Cl 1.10–2.03; *p* < 0.05) of having osteoporosis than their counterparts.
Kim MH et al., 2016 [50] South Korea	Osteoporosis (N = 244 men and 968 female) Healthy control (N = 1382 men and 453 female) 50 years old and over	24-h recall	BMD (no specific location)	Higher VC intake levels were associated with a lower risk of osteoporosis; the result was statistically significant (OR = 0.67; 95% CI: 0.47–0.97; *p* = 0.0371) only in the highest intake quartile group. No association was seen in the group with high physical activity. Significantly lower VC intake (81 ± 2.1 vs. 113.1 ± 2.0 mg/d; *p* < 0.05) was noted in the osteoporosis patients group.
Kim DE et al., 2016 [53] South Korea	189 postmenopausal women aged 60.63 ± 6.39 years old	FFQ 103 food items	LS, FN and TH BMD	T-score of the LS, FN and TH was positively correlated with intake of VC (r^2^ = 0.157, *p* = 0.048; r^2^ = 0.324, *p* < 0.001; r^2^ = 0.182, *p* = 0.003, respectively).
Casale M et al., 2016 [66] New Zealand	175 post-menarcheal and pre-menopausal women 16–45 years old	FFQ 220 food items	TB BMD	There was no relationship between VC intake and total body BMD.
Melaku YA et al., 2017 [64] Australia	1135 participants (N = 520 men, 615 female) 50 year old and over	FFQ 167 food items	BMD (no specific location)	Three nutritional patterns: mixed (potassium, calcium, fibre, retinol and vitamin B12); animal-sourced (cholesterol, protein, vitamin B12 and fat) and plant-sourced (fibre, carotene, VC and lutein). Whereas animal- and plant-sourced nutrient patterns were not associated with BMD, mixed source pattern may have had a beneficial effect on BMD reduction prevention.

BMD—bone mineral density, CT—computer tomography, N—number, TB-total body, LS—lumbar spine, FN—femoral neck, TH—total hip TF—total femur, FFQ—Food Frequency Questionnaire, PTH—parathyroid hormone, AP—alkaline phosphatase, VC—vitamin C, VE—vitamin E.

**Table 2:** Case control studies assessing the association between dietary vitamin C intake or vitamin C serum concentration and BMD or low energy fracture.

Study	Study Population	Dietary Assessment	Outcome Measure(s) and Lab Analyses	Results
Lumbers M. et al., 2001 [77] UK	75 women with femur neck fracture 80.5 ± 11.9 years old (range 61–103) 50 age-matched independent-living group of females attending one of three local day centres 79.8 ± 7.5 years old (range 63–95)	24-h dietary recall	Serum VC level	Plasma VC in fracture patients was significantly higher (7.52 ± 3.77 mg/L) than in control group (3.66 ± 2.50 mg/L *p* < 0.001). Daily intake of VC in diet was higher in group with hip fracture (60.7 ± 33.2 mg/d) than in control group (55.2 ± 38.8 mg/d) but it was not statistically significant.
Maggio D et al., 2003 [82] Italy	75 osteoporotic (T-score ≤ −3.5) 75 controls (T-Score ≥ −1) postmenopausal women Age over 60 years old	Mini Nutritional Assessment questionnaire	FN BMD Plasma VC level	FN BMD showed a positively statistically significant correlation with plasma VC (r = 0.26, *p* = 0.05). Plasma VC was significantly lower in osteoporotic group than in control (30 ± 3.7 vs. 55.5 ± 13.1 µmol/L; *p* < 0.001).
Martínez-Ramírez MJ et al., 2007 [71] Spain	Research group: N = 167, aged ≥ 65, osteoporotic fracture in 6–24 months prior to inclusion in the study Control group: N = 167	FFQ 136 food items	Plasma VC level	Daily dietary VC intake was higher in group with hip fracture (283 ± 12.8 mg/d) than in control group (263 ± 9.9 mg/d), but it was not statistically significant. Plasma VC in bone fracture patients was significantly lower (3.1 ± 0.3 mg/L) than in the control group (4.1 ± 0.3 mg/L; *p* = 0.012). For both dietary intake and serum levels, the lowest quartile of VC was associated with the highest risk of fracture. VC serum levels presented a linear trend (*p* = 0.03) with a significantly reduced fracture risk for the upper quartile (OR = 0.31; 95% CI 0.11–0.87) compared with the lowest quartile.
Park HM et al., 2011 [76] South Korea	72 osteoporotic women 72 controls (range 50–70 years old)	FFQ 117 food items	LS, FN and TF BMD	A significant reduction in the risk of osteoporosis for third quartile (136.9–176.3 mg/d) versus the lowest daily dietary VC intake (≤91.5 mg/d) was found. The correlation between VC intake and femoral neck BMD was presented (r = 0.190; *p* < 0.05)
Sun et al., 2014 [81] USA	726 elderly with hip fracture and 726 control subjects	FFQ 79 food items	None	The OR of hip fracture for the highest (>167 mg/d for men and >171 mg/d for women) vs. the lowest (<55 mg/d for men and <49 mg/d for women) quartile of VC intake was 0.39 (95% CI 0.28, 0.56). Daily dietary VC intake was significantly lower in both men and women with osteoporotic fracture (M: 77 ± 40 mg/d; F: 82 ± 47 mg/d)) compared to healthy control subjects (M: 102 ± 60 mg/d *p* < 0.001; F: 106 ± 57 mg/d *p* < 0.001).

LS—lumbar spine, FN—femoral neck, TF—total femur, FFQ—Food Frequency Questionnaire, VC—vitamin C, VE—vitamin E.

**Table 3:** Prospective and longitudinal studies assessing the association between dietary vitamin C intake or vitamin C serum concentration and BMD or low energy fracture.

Study	Study Group	Follow-Up in Years	Dietary Assessment	Outcome Measure(s) and Lab Analyses	Results
Kaptoge S et al., 2003 [84] UK	470 women and 474 men mean age 72 (range 67–79 years old)	2–5	7-day food diaries	TH BMD performed twice an average of 3 years apart	Women in the lowest tertile of dietary VC intake (7–57 mg/d) lost BMD at a faster rate compared to those in the middle (58–98 mg/d) (*p* = 0.015) and upper (*p* = 0.010) tertiles (99–363 mg/d). No relationship between VC intake and BMD loss was observed in men.
Macdonald HM et al., 2004 [75] UK	891 women aged 45–55 years old at baseline	5–7	FFQ 98 food items	LS and FN BMD at baseline and the end of observation	In premenopausal women, calcium intake and dietary fruit and vegetable intake were associated with FN BMD.
Sahni S et al., 2008 [72] USA	334 men and 540 women mean age 75 years old	4	FFQ 126 food items	BMD of right femoral neck and trochanter, lumbar spine, radial shaft at baseline used dual-photon absorptiometer; in follow-up used dual X-ray absorptiometry	No significant effects of VC intake on BMD in women was observed. LS and T BMD loss was significantly lower in smoking men with higher dietary VC intake. FN and T BMD loss was significantly lower in men with higher total VC intake, lower calcium and total VE intake.
Sahni S et al., 2009 [74] USA	366 men and 592 women, mean age 75 ± 5 years old In the 17 years of follow-up, 100 hip fractures and in the 15 years of follow up 180 non-vertebral osteoporotic fracture were reported among 976 participants	15–17	FFQ 126 food items	Clinical report of non-vertebral osteoporotic fractures	The group characterized by the highest tertile of total VC intake had a significantly lower risk of hip and non-vertebral osteoporotic fractures compared to subjects in the lowest tertile. Subjects in the highest category of supplemental VC intake had significantly lower risk of hip and non-vertebral osteoporotic fracture compared to non-supplementing study participants. A protective trend was observed for dietary VC and risk of hip fracture as well as non-vertebral osteoporotic fractures.
Sugiura M et al., 2016 [70] Japan	187 post-menopausal female Mean age round 60 *	4	FFQ 97 food items	Non-dominant forearm BMD measured at baseline and follow up. Concentration of 6 serum carotenoids	High VC intake with high serum concentration of β-cryptoxanthin was inversely associated with osteoporosis development risk.

* Information about whole group mean age was not included. BMD—bone mineral density, VC—vitamin C, VE—vitamin E, FFQ—Food Frequency Questionnaire, TH—total hip, LS—lumbar spine, FN—femoral neck, T—total.

**Table 4:** Interventional studies assessing the association between dietary vitamin C intake or serum vitamin C concentration and BMD or low energy fracture.

Study	Study Group	Follow-Up	Intervention	Outcome Measure(s) and Lab Analyses	Results
Bjarnason NH et al., 2001 [83] Denmark	68 postmenopausal women 65 years old and above; hip and/or spine BMD < −2.0 SD; serum cholesterol < 5.2 mmol/L	12 weeks	2 groups: 1. VC 500 mg/d; N = 23 2. 40 mg Fluvastatin + VC 500 mg/d; N = 45	Biochemical bone marker measurements (AP, OC, U-CTX, S-CTX) at visits –2, 0, 4 and 12 weeks.	No effect of the treatments on the markers of bone formation.
Chavan SN et al., 2007 [90] India	75 osteoporosis patients age 45–70 years and 50 healthy controls age 20–50 years (did not undergo the intervention) *	13 weeks	3 groups: 1. VE 400 mg/d; N = 25 2. VC 500 mg/d; N = 25 3. VE 400 mg/d + VC 500 mg/d; N = 25	Serum concentrations of malondialdehyde, glutathione peroxidase, glutathione reductase, superoxide dismutase, Ca^+^, Pi measured at baseline, 45th day and at the end of the study.	A significant fall in the concentration of serum malondialdehyde (*p* < 0.001), TrACP (*p* < 0.01) in all supplemented groups. Antioxidant status was reflected by significant rise in concentration of serum superoxide dismutase (*p* < 0.001) and erythrocyte GSH (*p* < 0.001) after 90 days of antioxidant supplementation in osteoporosic patients.
Maïmoun L et al., 2008 [68] France	9 postmenopausal women and 4 men 69–79 years old	8 weeks	VC 500 mg/d + vitamin E 100 mg/d + aerobic training programme	Serum concentration of ionized calcium, PTH, 25-hydroxyvitamin D, 1,25-dihydroxyvitamin D, osteocalcin, bone AP, urinary type I collagen, C-telopeptide, IGF-1 and IGFBP-3	After 8 weeks vitamin d level and bone alkaline phosphatase was statistically significantly higher, rest serum markers stayed constant. Intervention combining VC and VE supplementation combined with aerobic training might improve the calciotropic hormone profile which is altered in the elderly and associated with bone loss.
Chuin A et al., 2009 [88] Canada	34 postmenopausal women 61–73 years old	6 months	3-day dietary record Four groups: 1. Placebo; N = 7 2. Antioxidant (VE 600 mg/d + VC 1000 mg/d); N = 8 3. Exercise (60-min exercise sessions 3 x/week) + placebo; N = 11 4. Exercise + antioxidants; N = 8	BMD of FN and LS at baseline and at the end of the study.	The placebo group displayed a significant loss in LS BMD (*p* < 0.05) over 6 months; no changes observed in the other groups.
Ruiz-Ramos M et al., 2010 [85] México	90 elderly participants Group 1: 67.6 ± 7.3 years old; 66% female Group 2: 68.2 ± 7.3 years old; 87% female Group 3: 68.8 ± 8.5 years old; 68% female	1 year (follow-up 0, 3, 6, 9, 12 months)	Divided into 3 groups: 1. Placebo; N = 30 2. VC 500 mg/d + alpha-tocopherol 400 IU/d; N = 30 3. VC 1000 mg/d + alpha-tocopherol 400 IU/d N = 30	BMD of hip and spine at baseline and at the end of the study; Serum measurements of thiobarbituric acid reactive substances, total antioxidant status, superoxide dismutase, and glutation peroxidase	Differences observed in the hip BMD between the treatment groups and placebo, indicating a possible beneficial effect of antioxidants as a coadjuvant in the prevention and treatment of osteoporosis.
Masse PG et al., 2010 [73] Canada	60 women with osteopenia 35–55 years old, non-oestrogen users	1 year	3 non-consecutive days food recall; 3 groups: 1. Placebo, normal BMD; N = 20 2. Osteopenic, calcium 1000 mg/d + vitamin D3 250 IU/d; N = 20 3. Osteopenic, calcium 1000 mg/d + vitamin D3 250 IU/d + VC 500 mg/d + vitamin B6 75 mg/d + prolin 500 mg/d; N = 20	BMD of LS and femoral sites at baseline and at the end of the study. Blood sample: intact PTH, estradiol, calcium and inorganic phosphorus, OC, bone AP, vitamin D3 and B6. Urinary free deoxypyridinoline, type I collagen helical peptide, creatinine, vitamin B6 metabolites.	Osteopenic patients treated with the conventional calcium/vitamin D supplement continued to lose bone minerals to a much greater extent than the normal BMD control participants. The combination of calcium/vitamin D with collagen-related nutrients deterred further bone loss at all bone sites. Markers of bone turnover decreased significantly in both osteopenic groups. Although biomarkers of resorption did not change, PTH and 1,25(OH)_2_D_3_-induced osteoclastic activity were significantly reduced.
Mainini G et al., 2012 [87] Italy	44 postmenopausal women with osteopenia (49–75 years old)	1 year	2 groups: 1. alpha lipoic acid 300 mg + VC 30 mg + VE 5 mg + selenium 2.75 mg + calcium 500 mg + vitamin D3 400 IU twice a day; N = 23 2. calcium 500 mg + vitamin D3 400 IU twice a day; N = 21	BMD of non-dominant foot at baseline and after 1-year follow-up; heel quantitative ultrasonometry	The treatment with alpha lipoic acid led to a significantly higher estimated BMD compared to the control group (*p* = 0.048).
Stunes AK et al., 2017 [86] Norway	35 men age 68 ± 6 years old	12 weeks	4-weekday dietary registration 2 groups: 1. Placebo + strength training program n = 18 2. VC 1000 mg/d, VE 235 mg/d + strength training program	BMD of the LS, TH, FN, TB at baseline and after 12 weeks. Blood concentration of vitamins C, E, D; CTX-1. P1NP, osteoprotegerin, intact OC, PTH, insulin, sclerostin, DKK1, TNF-α, leptin, adiponectin, resistin, RANKL	High doses of VC + VE supplementation blunted some of the positive skeletal effects from strength training. Twelve weeks of resistance training intervention increased the LS and TH BMD. In the control group, physical activity increased TH and LS BMD, insulin-like growth factor and leptin concentrations. This effect was not present in the antioxidant supplementation group.

* No information about sex structure and mean age of study groups. TH—total hip, FN—femur neck, TB—total body, LS—lumbar spine, PTH—parathyroid hormone, DKK1—Dickkopf-related protein 1, VC—vitamin C, VE—vitamin E, AP—alkaline phosphatase, OC—serum osteocalcin, U-TX—urinary CrossLaps, S-TX—serum CrossLaps, PTH—parathyroid hormone, IGF-1—insulin-like growth factor 1, IGFBP3—insulin-like growth factor binding protein 3, CTX-1—C-terminal telopeptide from type 1 collagen, P1NP—procollagen type 1 N terminal propeptide, TNF-α—tumor necrosis factor α, RANKL—receptor activator for nuclear factor κB ligand.
